# Cross-Modal Transfer Learning From EEG to Functional Near-Infrared Spectroscopy for Classification Task in Brain-Computer Interface System

**DOI:** 10.3389/fpsyg.2022.833007

**Published:** 2022-04-07

**Authors:** Yuqing Wang, Zhiqiang Yang, Hongfei Ji, Jie Li, Lingyu Liu, Jie Zhuang

**Affiliations:** ^1^Shanghai Yangzhi Rehabilitation Hospital (Shanghai Sunshine Rehabilitation Center), Collage of Electronic and Information Engineering, Tongji University, Shanghai, China; ^2^Department of Neurorehabilitation, Shanghai Yangzhi Rehabilitation Hospital (Shanghai Sunshine Rehabilitation Center), School of Medicine, Tongji University, Shanghai, China; ^3^School of Psychology, Shanghai University of Sport, Shanghai, China

**Keywords:** fNIRS signals, transfer learning, brain computer interface, ICA, RCSP

## Abstract

The brain-computer interface (BCI) based on functional near-infrared spectroscopy (fNIRS) has received more and more attention due to its vast application potential in emotion recognition. However, the relatively insufficient investigation of the feature extraction algorithms limits its use in practice. In this article, to improve the performance of fNIRS-based BCI, we proposed a method named R-CSP-E, which introduces EEG signals when computing fNIRS signals’ features based on transfer learning and ensemble learning theory. In detail, we used the Independent Component Analysis (ICA) algorithm for the correspondence between the sources of the two signals. We then introduced the EEG signals when computing the spatial filter based on a modified Common Spatial Pattern (CSP) algorithm. Experimental results on public datasets show that the proposed method in this paper outperforms traditional methods without transfer. In general, the mean classification accuracy can be increased by up to 5%. To our knowledge, it is an innovation that we tried to apply transfer learning between EEG and fNIRS. Our study’s findings not only prove the potential of the transfer learning algorithm in cross-model brain-computer interface, but also offer a new and innovative perspective to research the hybrid brain-computer interface.

## Introduction

The brain-computer interface (BCI) is a communication way between the brain of users and the outside world, which can be used to exchange information between the brain and the equipment (Khosrowabadi et al., 2011). It has been employed in many fields, such as neuronal rehabilitation (Daly and Wolpaw, 2008), military (Rebsamen et al., 2007), traffic (Yue and Wang, 2019), and entertainment (Royer et al., 2010). In BCI, motor imagery is the most investigated paradigm. Many previous studies have demonstrated that the motor imagery brain-computer interface system (MI-BCI) has a broad prospect in rehabilitation training and neural recovery (Pichiorri et al., 2015; Bundy et al., 2017).

Among most studies about the MI-BCI system, researchers mainly focus on EEG signals, which record neural activity in the brain (Wolpaw et al., 2006). Considering its non-invasiveness and high temporal resolution (milliseconds), EEG is the most popular brain signal and provides encouraging results. fNIRS is a functional brain imaging tool, which reflects the activation degree of the cerebral cortex by measuring the level of oxygen in the blood (Ferrari and Quaresima, 2012). In contrast to EEG, fNIRS provides better spatial resolution and shows better tolerance to motion artifacts. The fNIRS-based BCI was mentioned firstly by Coyle et al. (2004). With the development of brain science and breakthroughs in cognitive neuroscience technology, fNIRS has developed rapidly as a tool to study cognitive neuroscience in the past decades.

However, as we know, due to the lower time resolution and lack of feature extraction algorithms, the fNIRS-based BCI system usually has poor performance. Common Space Pattern (CSP) is an effective method to extract features in the EEG-based BCI (Spuler et al., 2014; Wu et al., 2017). However, fNIRS signal is different from EEG. For EEG, it is a signal reflecting brain electrophysiology with rich high-frequency fluctuations while fNIRS is a slowly changing signal detecting cerebral hemodynamic response. Therefore, applying the CSP algorithm to fNIRS signals directly usually leads to poor effect. In article (Zhang et al., 2017), CSP algorithm was utilized to integrate multi-channel fNIRS signals into a single temporal sequence with the usage of an optimal spatial filter and subsequently, variance was extracted as part of the feature vector for brain state classification. They reported this method can improve the classification accuracy more than 9% in average. In our research, to improve the CSP algorithm in fNIRS, we introduced EEG signals based on the theory of transfer learning.

In addition, because fNIRS relies on cerebrovascular dynamics, the response is not as sensitive as EEG. Taking motor imagery as an example, the change of hemodynamic concentration is delayed relative to the start and end of the movement lasting about 5 s (Lachert et al., 2017). This results in the fact that it takes longer than EEG to collect a certain amount of data during the experimental session. Considering that the experiment time is too long, the subjects will be tired, which also limits the classification accuracy and application of fNIRS-BCI. Previous studies often used deep learning technology to improve classification accuracy. In the article (Trakoolwilaiwan et al., 2017), a convolutional neural network (CNN) was utilized to automatically extract features and classify in left or right-hand motor execution task. Results showed that the CNN-based methods improve the classification accuracy compared to traditional methods. Another popular approach is integrating the fNIRS signals with the EEG signals. EEG is one of the electrophysiological signals, and fNIRS measures the level of oxygen in the blood. Hence, combining the two signals could offer us different types of data that involved the same brain activity. Experimental results revealed that the fusion method could indeed enhance the performance of BCI (Fazli et al., 2012; Lee et al., 2019).

Recently, transfer learning (TL) has been widely discussed in the BCI system and has gradually become a new research hotspot (Jayaram et al., 2015). The existing methods are mainly applied in the EEG brain-computer interface system, which promotes learning a new subject/session/equipment/task by utilizing the data or knowledge from similar or relevant subjects/sessions/equipment/tasks (Wu et al., 2022). It dramatically solves the dilemma of small training data, significant individual differences, and few executable commands. Transfer learning is currently the most crucial method to reduce calibration in BCI. In this article (Khazem et al., 2021), a transfer learning approach called MDWM was proposed, which aims to reduce calibration time by transferring knowledge from other subjects to the target user. Similarly, this paper described a multi-source fusion transfer learning algorithm (Liang and Ma, 2020). However, there is little research in the multimodal brain-computer interface that involved the thought of transfer learning.

In this paper, to achieve TL between EEG and fNIRS, we proposed a method called R-CSP-E based on the Regularized Common Spatial Pattern (RCSP) theory, which states that the weighted covariance matrix can be transferred from EEG to fNIRS. RCSP, as a variant of the CSP algorithm, is used for cross-subject adaption (Xu et al., 2019). EEG signals and fNIRS signals were recorded simultaneously under the same thinking task activity. We believe that there is a certain amount of information between the two signals that can be transferred and utilized. Besides, our method also embodies the idea of ensemble learning. The results of multi-experiments showed that our method could recognize the characteristic patterns of the brain better and improve the classification accuracy of motor imagination tasks.

In summary, the contribution of our paper lies in the following three aspects. Firstly, we use the ICA algorithm to trace the source and find the region with the highest activation level to establish the correspondence between channels. Secondly, inspired by the thought of TL, we consider the features of the EEG signals to improve the performance of the brain-computer interface system based on fNIRS. Finally, we provide a new perspective to research the hybrid brain-computer interface.

The remainder of the sections of our paper is organized as follows: section “Materials and Methods” describes the dataset and methodology used in the experiment. Section “Experiments” is devoted to showing the experimental design and results, and sections “Results” and section “Discussion” are the discussion and conclusion of this article.

## Materials and Methods

### Dataset

**FIGURE 1:**
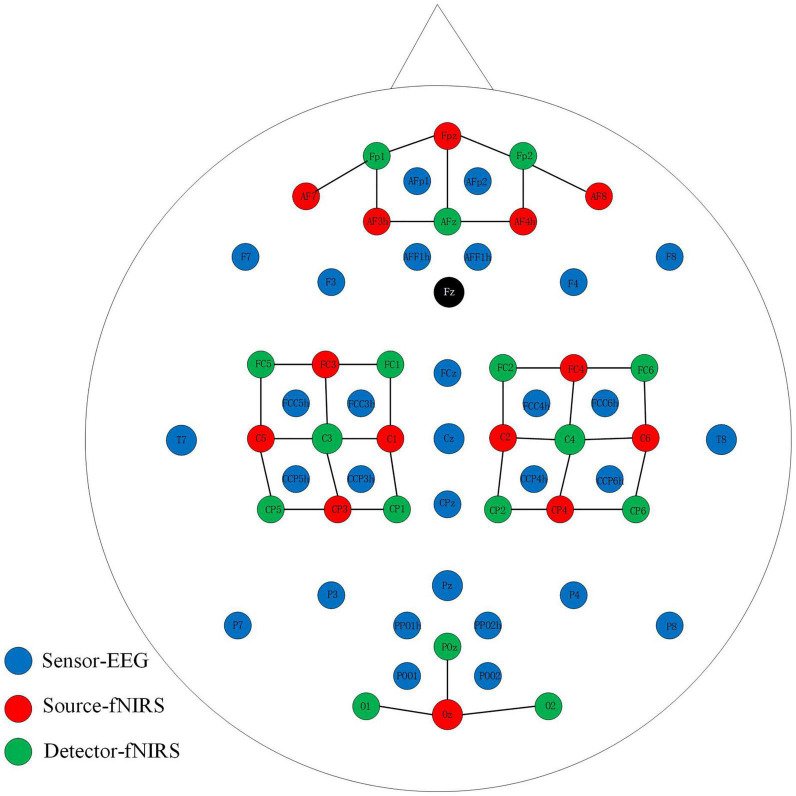
Placement of EEG electrodes, NIRS sources and detectors. The solid black line represents the NIRS channel.

An open-access dataset for EEG-fNIRS hybrid brain-computer interface with two brain activities, motor imagery (MI) and mental arithmetic (MA), was involved in our research (Shin et al., 2017). In our work, we mainly made full use of the MI dataset. For the EEG, the signals were recorded at 1000 Hz. As shown in Figure 1, thirty EEG active electrodes were placed according to the international 10-5 standard (AFp1, AFp2, AFF1h, AFF2h, F3, F4, F7, F8, FCz, FCC3h, FCC4h, FCC5h, FCC6h, T7, T8, Cz, CCP3h, CCP4h, CCP5h, CCP6h, CPz, Pz, P3, P4, P7, P8, PPO1h, PPO2h, POO1, POO2, and Fz for ground electrode) (Oostenveld and Praamstra, 2001). For fNIRS, fourteen sources and sixteen detectors represented thirty-six channels with a 12.5 Hz sampling rate. The detail and specific placement of electrodes can refer to this article (Shin et al., 2017). In general, placed channels near the frontal, motor, and visual areas. In order to realize the synchronous acquisition of signals, the fNIRS optodes and EEG electrodes were fixed on the same fabric cap.

**FIGURE 2:**
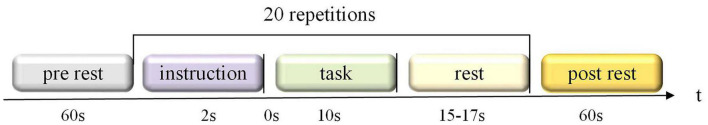
Task sequence of experimental paradigm.

All subjects sat on a chair 50 inches from the screen and asked to remain still and relaxed. Participants were instructed to execute left or right-hand squeezing imagery (i.e., to imagine the finger’s movement up and down in the brain) by visual stimulation. Each subject performed three sessions (each session contains twenty trials) of left or right-hand MI tasks. As is illustrated in Figure 2, a single trial consisted of instruction (2s), task (10 s), and rest (15 task (periods. The left or right arrow was displayed on the computer screen during the instruction.

### Pre-processing

All data processing was completed by MATLAB R2019b, and the BBCI toolbox was also applied for further data processing (Benjamin et al., 2010). The original EEG signals were re-referenced firstly and filtered with a passband of 0.5–50 Hz. When we collect EEG signals, there will be some unavoidable noises, such as electrooculogram (EOG), electromyogram (EMG). These signals will cause interference to our brain electricity. Hence, in data processing, these interference signals need to be eliminated. Then we performed the independent component analysis (ICA)-based artifact rejection to remove the ocular artifacts. This method assumes that the observed signals are composed of clean EEG data and independent noise components.

Down-sampled the EEG signals to 200 Hz and filtered between 8 and 30 Hz. Then normalized by Z-score and segmented into epochs ranging from −3 to 7 s. After that, subtract the mean value of the period ranging from −3 to 0s to correct the baseline. Electrodes attached to the motor cortex (FCC5h, FCC6h, FCC3h, FCC4h, CCP5h, CCP6h, CCP3h, CCP4h) were selected for further analysis.

Raw fNIRS signal is filtered at 0.01–0.1 Hz to remove internal noise generated by physiological activities such as respiration and heartbeat. After that, the filtered data are converted to a concentration of deoxy- and oxy-hemoglobin (HbR and HbO) according to the modified Beer-Lambert law (Kocsis et al., 2006). Then, considering that the hemodynamic response of fNIRS is relatively slow, the HbO and HbR signals are divided into periods of 0–10 s. Finally, we conducted the baseline correction by diminishing the mean value from 0 to 3 s. Similar to EEG, twenty-four channels near the motor cortex were used for further NIRS data processing.

### Methods

**FIGURE 3:**
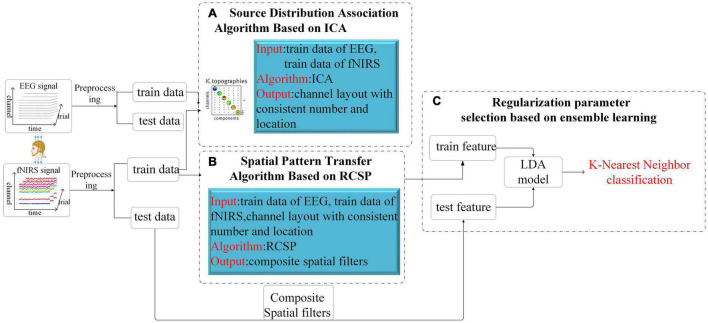
The total structure of the proposed Algorithm. After the EEG signal and the fNIRS signal are preprocessed respectively, they are divided into a training set and a test set. The training set data is adjusted by the ICA algorithm for channel order **(A)**, then the RCSP framework is used for feature generation **(B)**, and finally combined with LDA and KNN for classification **(C)**.

This part will introduce the overall framework of our algorithm and then describe the three essential components in detail. The whole scheme of our algorithm is shown in Figure 3, which consists of two parts. The first part is the model training. After preprocessing, the synchronously collected multimodal data are divided into the training and test datasets. After adjusting the channel through the proposed ICA-based source distribution association algorithm, the EEG training dataset is introduced into the calculation of the spatial filter and then constructed the composite spatial filters from the fNIRS data based on the RCSP framework. In RCSP, regularization parameter selection is crucial. In our study, this problem is solved by the idea of ensemble learning. Finally, the features extracted by the filter are input into Linear Discriminant Analysis (LDA) for dimensionality reduction, and K-nearest neighbors (KNN) is used for classification. Another part is the model testing. Test data is fed to the model to calculate the accuracy.

#### Source Distribution Association Algorithm Based on Independent Component Analysis

To realize the transfer between EEG and fNIRS signals, we first need to adjust the positions and numbers of the two signals’ channels to be consistent. Although EEG and fNIRS channels show one-to-many correspondence, which means that each EEG channel is associated with multiple fNIRS channels. But for the convenience of calculation, we need to make a one-to-one correlation. Therefore, this paper proposes channel matching from the perspective of source distribution. This section will introduce the channel adjustment strategy in detail. ICA is widely used as a popular technique to remove artifacts in the BCI field. In addition, for the motor imagery task, because the interstitial activity is significant and its source can usually be considered specific, the ICA algorithm is also suitable for extracting the exciting regions. ICA is a generative model, which describes how to generate observation data by mixing independent components (Hyvarinen and Oja, 2000). Considering the calculation speed, the ICA algorithm used in this article is FastICA (Dominic et al., 2010). In our study, we first obtain the source signal through the ICA algorithm. We believe that both EEG and fNIRS are based on motor imagery tasks, and the brain regions where the source signals generated have some certain consistency. We then matched channels by correlation of source signals.

**FIGURE 4:**
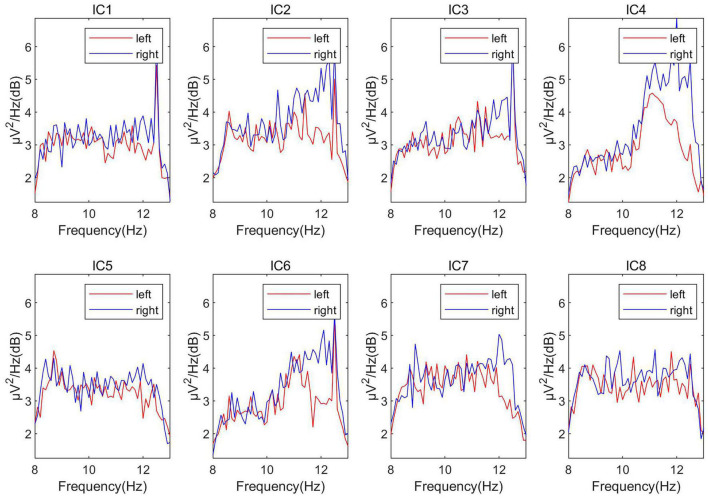
The power spectra of the independent components.

As described in Algorithm 1, we first used the ICA algorithm on the EEG signals to get eight independent sources (the number of channels is eight), which were marked as IC_1_∼IC_8_ in turn. Then, draw the power spectral density of these independent components. As shown in Figure 4, the power spectra of different independent components vary greatly. Calculate the distance between different types of power spectral density (PSD) curves on the power spectrum (the distance is calculated as the mean distance across all the frequencies). Then select the most obvious components that distinguish the left and right hands, and treat them as the most relevant source of classification, which is marked as S_eeg1_, S_eeg2_.

**Algorithm 1:** Source Distribution Association Algorithm Based on ICA.

**FIGURE 5:**
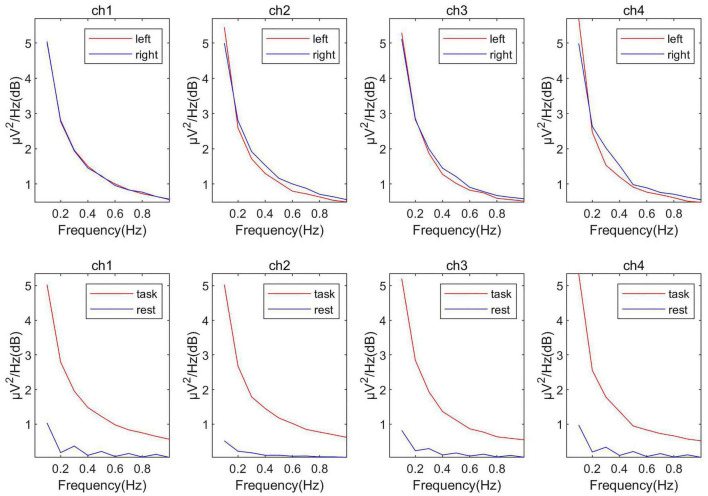
The power spectrum comparison of left and right handed and task-resting in fNIRS data.

**Input:** The multi-channel EEG signals, *E*_*n*_; The multi-channel fNIRS signals, *F*_*n*_;
**Output:** Channel layout with consistent number and location, *ch*;
1: Extract the set of independent sources of *E*_*n*_ based on ICA algorithm, named *IC*_*i*_;
2: Draw power spectral density on these independent components.
3: Select the two most obvious components that distinguish the left and right hands, which are marked as *S*_*eeg*1_, S*_*eeg*_*_2_
4: Extract the set of independent sources of *F*_*n*_;
5: Calculated the change of the source signals’ oxyhemoglobin concentration between the task period and rest period;
6: Pick up the two components with the largest variation. Denoted these two components as *S*_*fnirs*1_, *S*_*fnirs*2_;
7: Observe the columns in the mixing matrix belong to the four source signals, and sort the channels according to their weights;
8: **return** *ch*;


As shown in Figure 5, in the fNIRS data, using the left and right-handed tasks did not work because the response of the fNIRS signal was slower than that of the EEG signal. For the fNIRS signals, ICA decomposition was performed first and then calculated the change of the source signal’s oxyhemoglobin concentration between the task period and rest period. Like EEG, the two components with the largest variation were picked up as the most relevant sources of classification. Denoted these two components as S_fnirs1_, S_fnirs2_.

**FIGURE 6:**
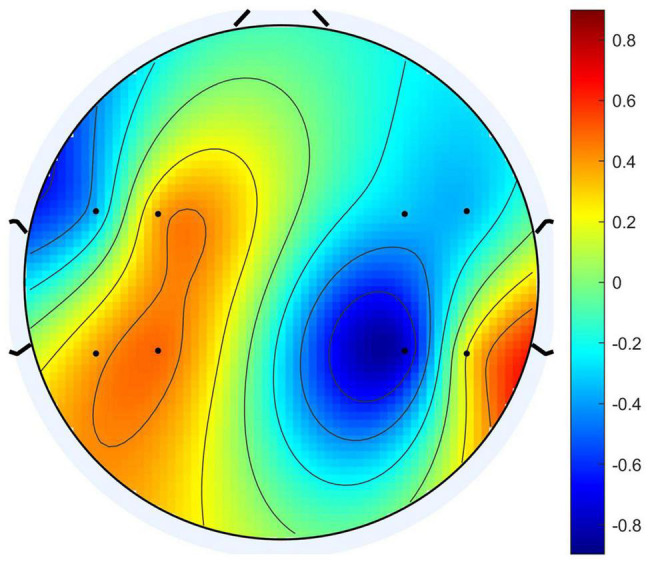
The pattern diagram of Seeg1.

Usually, when we compare the columns of the mixing matrix A in ICA, we can analyze how much the source data contributes to each channel of the observation data. Therefore, the columns of the mixing matrix can be used to calculate and visualize the topography of components. Motivated by the theory above, we observed the columns in the mixing matrix belong to the four source signals and sorted the channels according to their weights. For example, the pattern diagram of S_eeg1_ is shown in Figure 6. We sorted the channels according to the weight, the order of the channels is “FCC5h,” “FCC6h,” “CCP4h,” “CCP5h,” “FCC3h,” “FCC4h,” “CCP6h,” “CCP3h.” We deduced the channel sequence corresponding to the other three source signals by analogy. By combining them, the purpose of matching the EEG optode and the fNIRS channel was achieved.

#### Spatial Pattern Transfer Algorithm Based on Regularized Common Spatial Pattern

Common spatial patterns (CSP), which is regarded as the most effective feature extraction algorithm in the MI task, aims to extract the spatial distribution components of each class. The core of CSP is designing a set of spatial filters, thus the variance of one type of signal is maximized, and the other type is minimized, to obtain the eigenvectors with higher discrimination (Lu et al., 2009b).

Suppose X_1_ and X_2_ are the multi-channel signals during the motor imagery task, and their dimensions are both N × T (N is the number of channels, and T is the number of samples within each channel). The average spatial covariance matrix of each class can be expressed as Equation (1):


(1) $$\begin{array}{c} R_{1}=\frac{1}{k}*\sum_{i}^{k}\frac{X_{1}^{i}\,{\left(X_{1}^{i}\right)}^{T}}{t\,r\,a\,c\,e\,\left(X_{1}^{i}\,{\left(X_{1}^{i}\right)}^{T}\right)},R_{2}=\frac{1}{k}*\sum_{i}^{k}\frac{X_{2}^{i}\,{\left(X_{2}^{i}\right)}^{T}}{t\,r\,a\,c\,e\,\left(X_{2}^{i}\,{\left(X_{2}^{i}\right)}^{T}\right)} \end{array}$$

k represents the number of trials, X^T^ means the transposition of X, and trace (X) is the sum of the elements on the diagonal of the matrix. By calculating the average covariance matrix of each class separately, the composite spatial covariance is obtained.


(2) $$\begin{array}{c} R=R_{1}+R_{2} \end{array}$$

Then, according to Equation (3), the eigenvalue decomposition is performed on the composite spatial covariance matrix R.


(3) $$\begin{array}{c} R=U\,\lambda \,U^{T} \end{array}$$

Among them, U is the eigenvector matrix, and λ is the diagonal matrix composed of the corresponding eigenvalues. Rearrange the eigenvalues in descending order, and then obtained the whitening transformation.


(4) $$\begin{array}{c} P=\sqrt{\lambda^{-1}}\,U^{T} \end{array}$$

After the whitening transformation, R1 and R2 can be written as S1and S2, respectively.


(5) $$\begin{array}{c} S_{1}=P\,R_{1}\,P^{T},S_{2}=P\,R_{2}\,P^{T} \end{array}$$

Then, S1 and S2 can be factorized according to Equation (6), where λ1 and λ2 are the diagonal matrices composed of eigenvalues.


(6) $$\begin{array}{c} S_{1}=B_{1}\,\lambda_{1}\,B_{1}^{T},S_{2}=B_{2}\,\lambda_{2}\,B_{2} \end{array}$$

Through the above formula, it can be proved that the eigenvectors of matrix S_1_ and S_2_ are equal, namely:


(7) $$\begin{array}{c} B_{1}=B_{2}=V \end{array}$$

Meanwhile, the sum of λ_1_ and λ_2_ is the identity matrix. This attribute makes the eigenvectors B useful to distinguish the different classes.


(8) $$\begin{array}{c} \lambda_{1}+\lambda_{2}=I \end{array}$$

$\tilde{B}$ is concatenated by the first m and last m feature vectors in B (m is equal to 5). Next, calculate the projection matrix according to Equation (9). It contains 2m spatial filters.


(9) $$\begin{array}{c} W={\tilde{B}}^{T}\,P \end{array}$$

The feature vectors of the i_th_ trial as follows, where *var*(*Z*_*i*_) is the variance.


(10) $$\begin{array}{c} Z_{i}=W*X_{i},f_{i}=l\,o\,g\,\left(\frac{v\,a\,r\,\left(Z_{i}\right)}{sum(var\left(Z_{i}\right)}\right) \end{array}$$

Based on the theory of CSP and matrix regularization, researchers proposed the framework of RCSP to overcome the shortcomings of the traditional CSP model (Lu et al., 2009b). In our work, the regularized average spatial covariance matrix of each class is given as Equation (11).


(11) $$\begin{array}{c} {\hat{\Sigma }}_{c}\,\left(\beta ,\gamma \right)=\left(1-\gamma \right)\,{\hat{\Omega }}_{c}\,\left(\beta \right)+\frac{\gamma }{N}\,t\,r\,\left[{\hat{\Omega }}_{c}\,\left(\beta \right)\right]\cdot I \end{array}$$

where β and γ are both regularization parameters (0 ≤ β ≤ 1, 0 ≤ γ ≤ 1), and I is N × N identity matrix. ${\hat{\Omega }}_{c}\,\left(\beta \right)$concludes the covariance matrices from the fNIRS signals as well as EEG signals. It is defined as Equation (12).


(12) $$\begin{array}{c} {\hat{\Omega }}_{c}\,\left(\beta \right)=\frac{\left(1-\beta \right)\,R_{c}+\beta \,{\hat{R}}_{c}}{\left(1-\beta \right)\,M_{c}+\beta \,{\hat{M}}_{c}} \end{array}$$

where *R*_*c*_ is the sum of covariance matrices for all *M*_*c*_ training trials of the fNIRS signals in class c, and ${\hat{R}}_{c}$ is the sum of the covariance matrices for ${\hat{M}}_{c}$ generic training trials from EEG signals in class c. The composite spatial covariance in R-CSP is formed and factorized as Equation (13).


(13) $$\begin{array}{c} R={\hat{\Sigma }}_{1}\,\left(\beta ,\gamma \right)+{\hat{\Sigma }}_{2}\,\left(\beta ,\gamma \right)=U\,\lambda \,U^{T} \end{array}$$

In this study, we tried to apply this approach in transfer between EEG and fNIRS. the EEG signal is introduced according to Equation (12) when computing the spatial filter for fNIRS in the RCSP algorithm. The spatial pattern features of EEG are incorporated into fNIRS to realize the transfer of feature patterns.

#### Regularization Parameter Selection Based on Ensemble Learning

The critical problem of the RCSP algorithm proposed above is the determination of the regularization parameters. This question is crucial because it is challenging to know the optimal values of the parameters in advance. In earlier work, this problem is often solved through cross-validation. Our paper adopts the approach of ensemble learning for regularization parameter determination, named R-CSP-E. Ensemble learning refers to training multiple individual learners for training data, and finally we can form a powerful learner based on a certain combination strategy (Zhang and Ma, 2012). The commonly used combination strategy in regression tasks is the averaging method; the outputs of several weak learners are averaged to obtain the final predicted result. As for classification tasks, we usually use voting methods. The simplest voting method is the relative majority voting method, which is what we often say that the minority obeys the majority.

In R-CSP-E, a set of regularization parameter pairs from the interval [0,1] were utilized instead of applying fixed parameters. We recorded the results from different regularization parameters and combined them to form an aggregation solution, which reveals the theory of ensemble learning. Different regularization parameter pairs will produce different discriminative characteristics. Such diversity is conducive to training the classifier and improving the performance of the BCI system, based on the principle of boosting. The combination scheme originated from ensemble learning, which has been mainly developed in the following ways: feature, matching score, and ensemble-based learning, such as boosting (Lu et al., 2009a). In our study, the KNN classification algorithm was employed. This method embodies the idea of ensemble learning, but it is not ensemble learning in the strict sense. The principle of KNN is that if most of its K nearest neighbors in the training set belong to a specific category, the sample also belongs to this category. K is usually an odd number not greater than 20. In our algorithm, KNN is used as a classifier, and the feature dimension of training data (test data) is all 30 (the number of combinations of regularization parameters). For the test data, the distances to all dimensions of the training data are calculated and summed, and finally the labels of the k nearest training data to the test data are counted to complete the classification.

## Experiments

In this section, we carried out a set of experiments to investigate how the accuracy of fNIRS signals classification is affected by the EEG signals. As mentioned, the experiments were carried out on an open-access dataset. We only used the MI data, and the motor cortex was regarded as the region we were interested. Considering the differences between trials, we applied the five-fold cross-validation technique, which means that the training set of each iteration contains 48 trials, and the test set contains 12 trials. Besides, based on the study in Xu et al. (2019),we selected six values for β and five values for γ,which forms A = 6 × 5 = 30 different parameter combinations. This setting for R-CSP-E was used in all experiments in the following.


(14) β∈{0,0.01,0.1,0.2,0.4,0.6},γ∈{0,0.001,0.01,0.1,0.2}

Two experiments are executed as detailed in the following.

(1) To evaluate the effect of the R-CSP-E, we applied this method on EEG signals. R-CSP-E is a combination of cross-modal transfer learning and ensemble learning. In experiment I, only EEG data was involved, which means both *R*_*c*_ and ${\hat{R}}_{c}$ are EEG data in Equation (12). Hence, the results for experiment 1 showed the effectiveness of ensemble learning. We compared the accuracy of R-CSP-E with the conventional CSP algorithm as well as other classification algorithms, such as R-CSP-CV. In R-CSP-CV, the training set is divided into n parts, each part is used as a validation set in turn, and the remaining n-1 parts are used as a training set. In this way, for n times of training, the average value of the errors obtained in n times is taken as the final error of the model, so as to select the optimal model parameters. To investigate R-CSP-E, we also compared the impact of different values of k on the classification results.

(2) To study the transfer learning in EEG and fNIRS, we compared the correct classification rate of fNIRS before and after adding EEG signals. In detail, we calculated the mean value of the HbO concentration in each channel. Then, we extracted the features of channel-wise, conventional CSP algorithm and R-CSP-E separately.

In the above experiments, in R-CSP-E, we chose the LDA algorithm for dimensionality reduction. After LDA, the feature dimension is [number of trials] by [number of regularization parameters combinations]. KNN is used as the classifier. Finally, the average accuracy was shown, because we applied five-fold cross-validation. In other methods, such as CSP, KNN is also used as the classifier to make sure the classifiers are consistent. In R-CSP E, the input dimension for classification module is 60 × 30 (60 is the number of trials, 30 is the number of regularization parameter combinations). In R-CSP-CV and CSP, the input dimension for classification module is 60 × 10 (60 is the number of trials, 10 is 2 m). In HbO, the input dimension for classification module is 60 × 24 (60 is the number of trials, 24 is the number of channels).

In order to prove whether the effect of the R-CSP-E algorithm is significantly different, this paper uses the *t*-test (the full name is independent sample *t*-test) to study the difference in the accuracy of different algorithms for the results of experiment 2. The independent samples *t*-test is used to compare the means of two independent samples, which assumes that the difference between the two-sample means is equal to 0. Calculates the difference between two means and the confidence interval (CI) for that difference. Next follow the test statistic t, degrees of freedom (DF), and two-tailed probability P. When the *p*-value is less than the usual 0.05, the null hypothesis is rejected and it is concluded that the two means are indeed significantly different.

## Results

**FIGURE 7:**
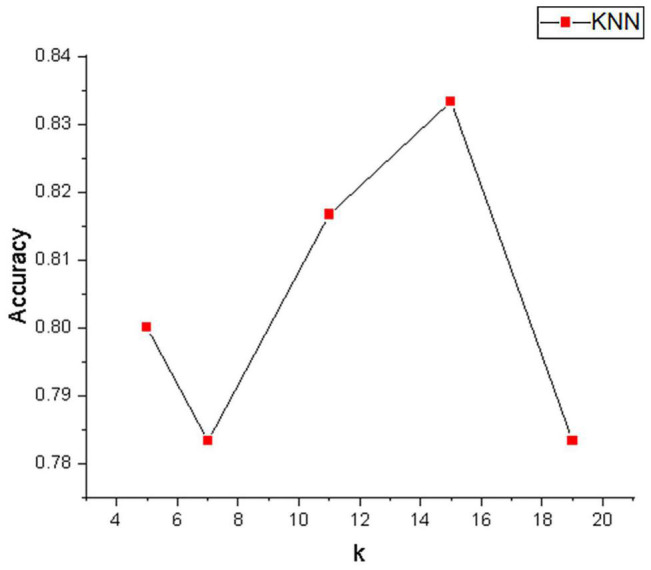
The average accuracy of classification on different *k*-value.

Table 1 depicts the EEG classification performance of experiment I. The overall average classification accuracy for CSP, R-CSP-CV, and R-CSP-E are 70.2, 72.2, and 78.2%, respectively. As shown in Table 1, in all participants, the R-CSP-E algorithm outperforms the traditional CSP algorithm, with an average improvement of 7% in the correct classification rate with respect to the conventional CSP method. This result demonstrates the effectiveness of the CSP regularization scheme and illustrates the advantages of the proposed ensemble learning method over traditional cross-validation. In addition, as shown in Figure 7, in the R-CSP-E algorithm, different values of k will lead to different results. When *k* = 15, the average performance is the highest. Therefore, in subsequent experiments, we set *k* = 15.

**TABLE 1:** Classification accuracy (%) of all subjects of different algorithms in experiment 1.

Subject	Algorithm		
	CSP	R-CSP-CV	R-CSP-E
1	83.3	78.3	85.0
2	65.0	71.7	78.3
3	75.0	73.3	83.3
4	71.7	75.0	76.7
5	66.7	65.0	75.0
6	63.3	66.7	70.0
7	71.7	75.0	81.7
8	68.3	68.3	75.0
9	75.0	71.7	78.3
10	80.0	78.3	83.3
11	71.3	75.0	78.3
12	76.7	78.3	83.3
13	63.3	65.0	70.0
14	71.7	76.7	81.7
15	68.3	73.3	75.0
16	71.7	78.3	85.0
17	55.0	61.3	73.3
18	81.3	78.3	86.7
19	68.3	70.0	78.3
20	66.7	71.7	68.3
21	71.7	78.3	83.3
22	58.3	61.3	65.0
23	73.3	65.0	75.0
24	65.0	76.7	81.7
25	75.0	71.7	80.0
26	81.3	85.0	91.7
27	71.7	73.3	80.0
28	56.7	55.0	63.3
29	68.3	75.0	81.7

The complete results for all subjects on the testing data set for experiment II are summarized in Table 2. In general, the classification rate of R-CSP-E showed superiority. Compared with the channel-wise feature extraction method and the conventional CSP algorithm, R-CSP-E can improve the classification accuracy up at least 6% on average. In order to clarify that the improvement of accuracy is derived from the effects of transfer learning or ensemble learning. The R-CSP-CV, which only includes effects of transfer learning, is tested for the fNIRS data. As depicted in Table 2, the result of R-CSP-E is better in the majority of subjects. This not only shows that the proposed R-CSP-E method is effective, but also concludes that the combination of the transfer learning and ensemble learning is crucial to improve accuracy. Besides, an independent *t*-test was performed to validate the outperformance of R-CSP-E over HbO, CSP and R-CSP-CV, separately.

**TABLE 2:** Classification accuracy (%) of all subjects of different algorithms in experiment 2.

Subject	Algorithm			
	HbO	CSP	R-CSP-E	R-CSP-CV
1	45.0	51.7	58.3	50.0
2	71.7	63.3	66.7	65.0
3	63.3	55.0	61.7	53.3
4	60.0	61.7	68.3	63.3
5	61.7	65.0	71.7	58.3
6	48.3	50.0	58.3	51.7
7	61.7	63.3	68.3	65.0
8	66.7	68.3	71.7	63.3
9	55.0	61.3	66.7	58.3
10	60.0	55.0	63.3	56.7
11	55.0	61.3	68.3	63.3
12	58.3	63.3	65.0	55.0
13	61.7	60.0	68.3	58.3
14	55.0	68.3	71.7	65.0
15	61.7	63.3	70.0	65.0
16	60.0	66.7	68.3	61.7
17	48.3	50.0	58.3	53.3
18	53.3	63.3	73.3	60.0
19	55.0	58.3	61.7	55.0
20	61.7	65.0	68.3	58.3
21	63.3	70.0	65.0	61.7
22	51.7	55.0	58.3	51.3
23	68.3	65.0	71.7	65.0
24	51.7	53.3	63.3	55.0
25	60.0	51.7	65.0	48.3
26	61.7	65.0	73.3	65.0
27	58.3	63.3	68.3	61.7
28	68.3	66.7	71.7	65.0
29	66.7	63.3	73.3	58.3

As shown in Tables 3–5, for mean value of R-CSP-E algorithm compared with that of HbO, it shows a higher accuracy with *p* = 0.00001 < 0.01, for mean value of R-CSP-E algorithm against that of CSP, higher accuracy is observed with *p* = 0.001 < 0.01, and for mean value of R-CSP-E algorithm against that of R-CSP-CV, higher accuracy is observed with *p* = 0.00001 < 0.01.

**TABLE 3:** Independent *t*-test between HbO and R-CSP-E.

	Algorithm (Mean ± SD)		*t*	*p*
	HbO (*n* = 29)	R-CSP-E (*n* = 29)		
Accuracy	59.08 ± 6.45	66.83 ± 4.81	−5.183	0.000**

*p < 0.05, **p < 0.01.

**TABLE 4:** Independent *t*-test between CSP and R-CSP-E.

	Algorithm (Mean ± *SD*)		*t*	*p*
	CSP (*n* = 29)	R-CSP-E (*n* = 29)		
Accuracy	60.94 ± 5.81	66.83 ± 4.81	−4.203	0.000**

*p < 0.05, ** p < 0.01.

**TABLE 5:** Independent *t*-test between R-CSP-CV and R-CSP-E.

	Algorithm (Mean ± SD)		*t*	*p*
	R-CSP-CV (*n* = 29)	R-CSP-E (*n* = 29)		
Accuracy	59.00 ± 5.21	66.83 ± 4.81	−5.946	0.000**

*p < 0.05, **p < 0.01.

**FIGURE 8:**
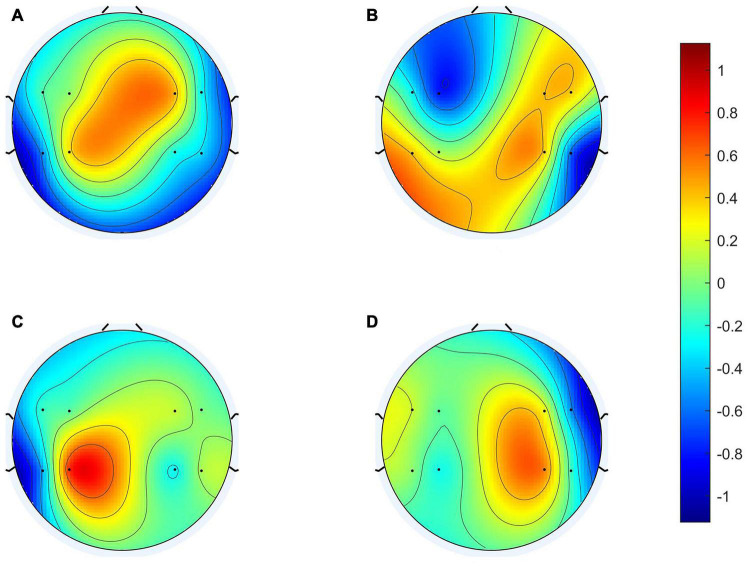
18th subject’s spatial patterns. **(A,C)** Are spatial patterns of LH, **(B,D)** are spatial patterns of RH. The spatial patterns in the first row are generated from Conventional CSP, and the spatial patterns in the second row are generated from our algorithm.

**FIGURE 9:**
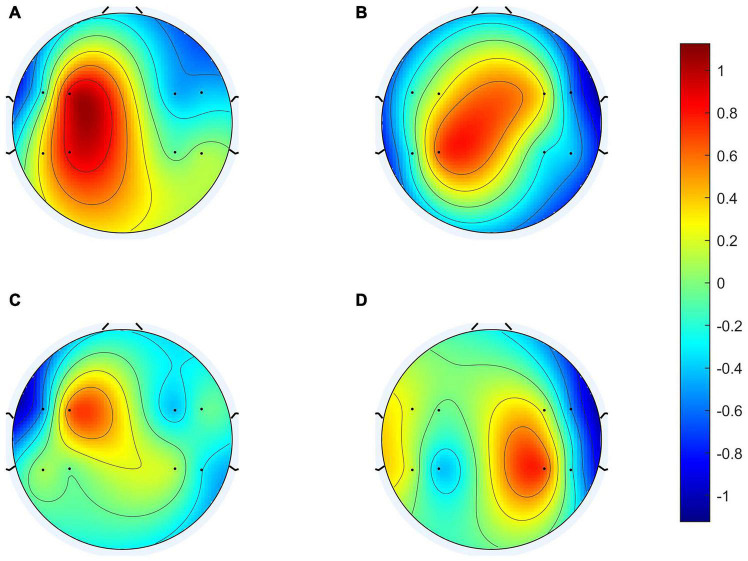
8th subject’s spatial patterns. **(A,C)** Are spatial patterns of LH, **(B,D)** are spatial patterns of RH. The spatial patterns in the first row are generated from Conventional CSP, and the spatial patterns in the second row are generated from our algorithm.

To clarify the characteristics of the transfer algorithm, we visualized and compared the spatial patterns generated by the traditional CSP algorithm and our R-CSP-E method. Figures 8, 9 are the most convincing of them, extracted from the 18th and 8th subjects, respectively. In both figures, the first row is CSP, and the second row is our algorithm. Applying traditional CSP and our algorithm to the 8th subject, the accuracy rates are 68.3 and 71.7%, respectively, which is relatively high. Hence, the optimization of our proposed algorithm has almost no improvement. As for the 18th subject, the accuracy is 63.3 and 73.3%, respectively. The CSP algorithm has a low accuracy rate, while the transfer algorithm achieves a significant increase in the classification rate, which can be explained by the spatial patterns. In Figure 7, the result reveals that our algorithm has a higher discrimination weight on the CP3, FC4, and CP6 channels.

The R-CSP-E filter is more discriminative than the CSP filter by introducing the EEG signal. More interestingly, it is expected from the neurophysiology literature. For example, for subject 18, the CSP filter appears to be rough and noisy, with greater weights at potentially different electrode positions, while the corresponding R-CSP-E filter shows greater weight in a few areas located on the motor cortex of the brain.

## Discussion

The main strength of our method is that the feature space of EEG signals is considered when computing the features of fNIRS signals. However, there are some limitations in this study that could be addressed in future research:

(1) At present, our transfer learning framework is based on the RCSP framework, which a simple but effective. In recent years, many more advanced feature extraction methods have been proposed successively, which can improve the classification performance by exploiting more spatiotemporal information (Lachert et al., 2017; Qi et al., 2020). Therefore, in future work, we can try to utilize these methods to further explore the application of transfer learning in cross-modal BCI.
(2) In our experiments, the regularization parameters are selected through the idea of ensemble learning. In the future, we will try more parameter selection methods.
(3) Currently, our cross-modal transfer learning is from EEG to fNIRS. However, in theory, transfer is mutual. In the future, we will further explore reverse transfer, the transfer of feature space from fNIRS to EEG.

## Conclusion

In summary, we proposed an algorithm named R-CSP-E based on the RCSP framework and transfer learning theory. Instead of simply fusing them, we innovatively applied transfer learning to the EEG-fNIRS multimodal brain-computer interface. Our outcomes showed that the R-CSP-E algorithm significantly improved the classification accuracy compared with conventional CSP and channel-wise methods in fNIRS.

As mentioned, MI-BCI has a broad prospect in rehabilitation training and neural recovery. fNIRS has also shown its potential in the investigation of functional brain activation patterns and neurorehabilitation. For example, a study evaluated the changes in cerebral cortex activation in stroke patients 2 months before and after rehabilitation (Arun et al., 2020). This study proved the potential of fNIRS in detecting changes in brain activation related to exercise recovery, which is reflected in the hemispheres affected after rehabilitation that increased activation of the premotor cortex. In this way, fNIRS has shown great potential as a neurorehabilitation tool to monitor the patient’s movement and cognitive improvement over time. In addition, fNIRS can also be used in the BCI system to treat movement disorders. Therefore, it is necessary to enhance the performance of fNIRS BCI, which will promote the development of cognitive neuroscience and neural plasticity.

## Data Availability Statement

Publicly available datasets were analyzed in this study. This data can be found here: http://doc.ml.tu-berlin.de/hBCI.

## Author Contributions

YW carried out experiment and writing. JL and HJ designed the overall framework. LL, JZ, and ZY contributed to methodological guidance and formal analysis. All authors contributed to the article and approved the submitted version.

## Conflict of Interest

The authors declare that the research was conducted in the absence of any commercial or financial relationships that could be construed as a potential conflict of interest.

## Publisher’s Note

All claims expressed in this article are solely those of the authors and do not necessarily represent those of their affiliated organizations, or those of the publisher, the editors and the reviewers. Any product that may be evaluated in this article, or claim that may be made by its manufacturer, is not guaranteed or endorsed by the publisher.
